# Highlighting person-centredness in technology supported care within nursing homes: a multiple case study

**DOI:** 10.3389/frhs.2026.1830120

**Published:** 2026-09-15

**Authors:** A. C. Noordam, C. J. Nierse, R. J. E. Vugs, V. T. J. Bisschops, J. Adriaansen, T. J. van der Zijpp, S. Cardiff

**Affiliations:** 1 Research Group Technology for Person-Centred Care, Department of People and Health Studies, Fontys University of Applied Sciences, Eindhoven, Netherlands; 2 Mijzo, Waalwijk, Netherlands; 3 Novicare, Best, Netherlands; 4 TanteLouise, Bergen op Zoom, Netherlands

**Keywords:** healthcare technology, long-term care, monitoring technologies, nursing homes, person-centred care (PCC)

## Abstract

Healthcare technologies are increasingly introduced in long-term care to support monitoring, assessment and clinical decision-making. In nursing homes, however, their use becomes intertwined with everyday care practices and relationships between professionals, residents and family members. This study explores how digital health technologies shape care relationships in nursing homes from a person-centred care perspective. Using the Person-Centred (Nursing) Practice Framework as an analytical lens, a secondary interpretive cross-case analysis was conducted based on three previously prepared narrative case reports describing technology use in different nursing homes in the Netherlands. The original case studies were reported in a narrative case format, providing contextualised insights into how technologies were enacted and experienced in everyday practice. The cross-case analysis explored patterns and variations in how technology-supported care was integrated within care relationships and organisational contexts. Across the cases, professionals and relatives described how timely information and monitoring provided reassurance and supported more responsive care. At the same time, working with technology-generated data required ongoing interpretation and contextualisation by professionals. Technology use was associated with changes in care routines and, in some instances, shifts in roles and responsibilities among professionals. Tensions between safety, privacy and autonomy were also identified, particularly where monitoring technologies were used in dementia care. These findings suggest that the value of technology-supported care does not lie in the technologies alone, but also in how they are introduced, interpreted and integrated within care relationships, professional practices and organisational contexts. Understanding technology-supported care as relational and context-dependent may help inform more person-centred integration of digital health technologies in nursing homes.

## 1 Introduction

Digital health technologies are increasingly promoted as part of the response to population ageing and rising care demands in long-term care settings. International policy bodies emphasize that information and communication technologies (ICT) can support older people in maintaining independence, health and active participation in society (1, 2). In line with these developments, the UN Decade of Healthy Ageing frames digitalisation as an important driver of innovation and efficiency in health and care systems (3).

At the same time, international organizations stress that the anticipated benefits of digital technologies depend on usability, accessibility and meaningful integration into these health and care settings (1, 4). However, current technology development for older people remains largely focused on functional healthcare applications (e.g., monitoring, diagnostics and risk detection), with relatively limited attention to the broader lived experience of ageing, such as social connectedness, autonomy and everyday wellbeing (2, 4).

From a person-centred perspective, this raises a critical consideration: not only whether technologies are adopted, but whether they are utilised in ways that align with the core person-centred values, including recognising the personhood of end-users, supporting their right to self-determination, and fostering mutual respect and understanding within care relationships (5, 6). Most research and implementation efforts continue to focus primarily on adoption processes, implementation outcomes and system efficiency. Comparatively little attention has been paid to how digital health technologies influence interpersonal care relationships and practices in everyday long-term care settings (7).

### 1.1 Practice context and rationale

This study was conducted in the Netherlands, where nursing home organisations are increasingly implementing digital health technologies to address rising care complexity, workforce pressure and demands for quality improvement (8, 9). Within long-term care for older people, nursing homes represent a key setting in which such technologies are becoming embedded in daily care. In this sector, person-centred care is also widely promoted as a guiding principle for quality of care (10, 11).

As technology use becomes more embedded in everyday practice, implementation is increasingly viewed as an ongoing organisational priority rather than a one-time innovation (12). Consequently, technology-supported care is becoming part of routine care and everyday interactions between professionals, residents and family members. Yet empirical insight into how such care is enacted from a person-centred perspective remains limited.

This paper presents a secondary interpretive cross-case analysis of three narrative case reports derived from practice-based qualitative studies conducted in Dutch nursing homes to explore how technologies are interpreted, mediated and integrated within care relationships in everyday practice.

### 1.2 Aim

Our aim was to explore how digital health technologies shape care relationships between professionals, residents and family members in nursing homes from a person-centred care perspective.

## 2 Methods

### 2.1 Design

This study used a secondary interpretive cross-case design. The analysis drew on previously prepared narrative case reports of digital health technology use in Dutch nursing homes, rather than on a systematic re-analysis of the original interview and observational datasets. Cross-case comparison enabled similarities, differences and context-specific variations across technologies and care settings to be explore (13).

### 2.2 Case origins and selection

The three cases originated from practice-based research projects conducted by master's students in the Advanced Nursing Practice programme at Fontys University of Applied Sciences, the Netherlands. Within this programme, students may participate in an academically supervised research group exploring the role of technology in person-centred care.

Cases were considered eligible for the present study when they: 1) involved the introduction or use of a relatively new digital health technology in nursing home care; 2) originated from a completed master's research project within the programme; 3) used a qualitative case study design; 4) included participant interviews; 5) incorporated perspectives from more than one stakeholder group; and 6) were reported in a narrative case format containing contextual descriptions and verbatim participant quotations. The inclusion of observational data was considered desirable because it provided greater contextual depth, but was not required for eligibility. Eligibility did require that the original student researcher and academic supervisor were available to participate in the secondary analysis, allowing contextual clarification and critical review of the interpretations.

Initially, two cases that included both interviews and observational data were identified. A third case was subsequently included to broaden the analytical basis and increase variation in technology type and care context. Although this case did not include observational data, it met all other eligibility criteria and provided interview data from multiple perspectives, together with contextual information, to support cross-case interpretation. Together, these cases provided variation in technology type and care context, allowing recurring patterns and context-specific differences to be examined (see Table 1 for an overview of the technologies used in each case). The three cases were:

- Case 1 (C1) - Vision-based safety monitoring (smart sensor) (14)
- Case 2 (C2) - Telehealth-enabled diagnostic assessment (remote diagnostics)
- Case 3 (C3) - Nocturnal sleep and physiological monitoring (bed-based sensor)

**Table 1:** Overview of included technology cases in Dutch nursing homes.

CASE ID	Case 1 (C1)	Case 2 (C2)	Case 3 (C3)
Technology type	Vision-based safety monitoring	Telehealth-enabled diagnostic assessment	Nocturnal sleep and physiological monitoring
Brief description	Camera-enabled contactless monitoring and alert system: Detects resident movement and bed-related activity using a ceiling-mounted camera, generating real-time safety alerts that enable care staff to remotely assess situations (e.g., potential falls) without entering the room.	Remote clinical assessment and diagnostic device: Enables nurses to perform physical assessments using the technology (e.g., heart and lung auscultation, temperature measurement, and throat or ear inspection) and the clinical data is then digitally transmitted for review by a remote practitioner.	Bed-based sleep and physiological monitoring system: The technology continuously monitors bed presence and movement, via an under-mattress sensor. Time-based insights into sleep patterns, night-time activity and physiological condition are reported.
Purpose & triggers	Fall risk detection and prevention Safety monitoring and alerting Applied to *all* residents as part of standard care.	Remote clinical diagnostics Clinical monitoring Resident, family and professional concerns about any acute changes in the resident's medical condition.	Sleep and night-time monitoring Detection of nocturnal unrest Reported disturbed sleep patterns.
Case environment	Nursing home (psychogeriatric ward)	Nursing home (somatic and psychogeriatric wards)	Nursing home (psychogeriatric ward)
Case resident	Residents with psychogeriatric conditions (e.g., dementia)	Residents with somatic and/or psychogeriatric conditions	Residents with psychogeriatric conditions (e.g., dementia)
(Standard) Decision-making procedure for the technology supported care	Implemented at organisational level as part of standard care provision; all residents and families are informed.	The care team decides, usually as a consultation between a nurse and a medical practitioner.	Initiated by the multidisciplinary team (e.g., nurse practitioner, psychologist or physician) during resident care reviews.
Brief description of technology use in daily practice	Nursing staff monitor alerts received via the technology and independently determine follow-up actions are required, based on their professional knowledge of the resident and the situation	Nurses perform the measurements, after which a medical practitioner evaluates the results and determines follow-up care	Time-based sensor data is primarily retrieved and trends interpreted by a nurse practitioner, psychologists and physician. Care staff tend to use the system more specifically and sporadically to check bed presence or night-time activity.
Evolution of technology usage	Implemented as part of routine care during the study period; later discontinued following internal evaluation.	Discontinued due to limited financial sustainability and absence of demonstrated workload reduction.	Implemented on all beds at the study location following the study; ongoing use highlighted challenges in integrating objective sensor data with contextual clinical information.

The included cases differed in participant composition, duration, data-collection procedures and procedures used to enhance quality and rigour (see Table 2), but all were conducted within the same master's programme. Within this programme, student researchers received comparable training in qualitative case study research methods and conducted their studies under academic supervision. All narrative case reports contained contextual descriptions of the care setting and technology use, together with participant accounts illustrated by verbatim quotations from care professionals, family members and, where available, residents.

**Table 2:** Methodological characteristics of the included case studies.

CASE ID	Case 1 (C1)	Case 2 (C2)	Case 3 (C3)
Technology	Camera-enabled contactless monitoring and alert system	Remote clinical assessment and diagnostic device	Bed-based sleep and physiological monitoring system
Original study aim	To explore user experiences and shared decision-making regarding the use of the technology	To explore stakeholder experiences with the technology and what is needed for person-centred integration into daily care	To explore how the technology contributes to the selection of appropriate interventions for sleep problems
Original study design	Case study	Case study	Case study
Data collection methods	Group/individual interviews Practice observations File analysis	Group/individual interviews Practice observations	Group/individual interviews File analysis
Participants	Total participants: *n* = 11 Care professionals (incl. care advisor and innovator) (*n* = 9) Family caregivers (*n* = 2)	Total participants: *n* = 17 Care professionals (*n* = 8) Organisational liaison (*n* = 1) Technology supplier (*n* = 1) Residents (*n* = 7)	Total participants: *n* = 13 Care professionals (*n* = 12) Family caregiver (*n* = 1)
Interviews	Total interviews: *n* = 12 Individual (*n* = 11) Group (*n* = 1)	Total interviews: *n* = 26 Individual (*n* = 24) Group (*n* = 2)	Total interviews: *n* = 18 Individual (*n* = 10) Group (*n* = 8)
Observations	Total observations: *n* = 9	Total observations: *n* = 19	Total observations: *n* = 0
Period of data collection	January – April 2024	November 2024 – March 2025	November 2023 – January 2024
Quality and rigour procedures	Data-source triangulation Peer review of coding and theme development Interview transcripts and observation notes returned to participants for verification Participant validation: narrative discussed and validated in a focus group	Data-source triangulation Peer review of coding and theme development Full individual member checking not completed Participant validation: preliminary findings discussed in a focus group Research log maintained for traceability and reflexivity	Data-source triangulation Peer review of coding and theme development Full individual member checking not completed Participant validation: not completed Research log maintained for traceability and reflexivity
Original analytical approach of data-analysis	Inductive thematic analysis (Braun & Clarke) Themes presented in a narrative supported by verbatim quotations	Inductive thematic analysis (Braun & Clarke) Themes presented in a narrative supported by verbatim quotations	Inductive thematic analysis (Braun & Clarke) Themes presented in a narrative supported by verbatim quotations
Source report	Published journal article in *Verpleegkunde* (14)	Unpublished master's report; retained by the original researcher	Unpublished master's report; retained by the original researcher

Note: For the cross-case analysis, the narrative case reports served as the source material; the original raw datasets were not systematically re-analysed. Residents were directly included as interview participants only in C2; consequently, resident perspectives were less represented across the other cases. Some participants were interviewed more than once; consequently, the number of interviews may exceed the number of individual participants.

### 2.3 Analytical framework

Analysis was guided by the Person-Centred (Nursing) Practice Framework (PC(N)PF) (5, 6), which conceptualises person-centred care across four interrelated domains: person-centred outcomes, person-centred processes, the care environment, and prerequisites. The PC(N)PF was used as a sensitising lens to guide the identification, interpretation and organisation of relevant material within the narrative case reports, rather than as a prescriptive coding template. This allowed for a theoretically informed yet flexible exploration of how technology-supported care practices and care relationships took shape in everyday nursing home care.

### 2.4 Data and analysis

The secondary analysis was based on the three narrative case reports containing contextual descriptions of the care settings and technology use, reported experiences, and selected verbatim quotations from care professionals and family members and, in C2, residents. The original interview and observational datasets were not systematically re-analysed, but the original student researchers and their supervisors were part of the team conducting this secondary analysis. Analysis followed an interpretive qualitative approach and proceeded in two stages: within-case analysis followed by cross-case comparison.

#### 2.4.1 Within-case analysis

First, each narrative case report was read and re-read to develop an in-depth understanding of the case context, the technology used and the perspectives represented. Second, fragments relevant to person-centred care were identified using the PC(N)PF as a sensitising lens. These fragments included contextual descriptions, reported experiences and verbatim quotations relating to care relationships and everyday technology-supported care practices. Third, the selected fragments were provisionally interpreted as expressions of perceived value in relation to person-centred care (claims) or as tensions and limitations (concerns). The initial interpretations were critically reviewed within the research team. Researchers involved in the original case studies subsequently provided contextual clarification where needed and checked whether the interpretations and translated quotations remained consistent with the original narrative case reports.

#### 2.4.2 Cross-case analysis

Following the within-case analysis, claims and concerns were compared across the three cases to identify recurring patterns, differences and context-specific variations in how technology-supported care was enacted. The identified fragments and interpretations were organised within the four domains of the PC(N)PF. Comparisons were made across cases while retaining attention to differences in technology and care context, rather than assuming that findings from one case applied to the others. The emerging cross-case synthesis was discussed within the wider research team and refined through critical peer dialogue.

Some members of the research team had been involved in the original case studies, whereas the first and last authors had not. This allowed the team to draw on both contextual familiarity and analytical distance when critically examining the emerging interpretations. Throughout the analysis, selected quotations and interpretations were checked against the narrative case reports to maintain consistency with the source material. Where quotations required translation from Dutch into English, translations were reviewed by authors fluent in both languages.

### 2.5 Ethical considerations

Ethical review followed applicable institutional or organisational procedures: two studies were reviewed by the ethics committee of the University of Applied Sciences, and one study received approval from the scientific review board of the participating care organisation, as well as the local site manager and client council. Participation was voluntary and informed consent was obtained from all participants or their legal representatives when residents had limited decision-making capacity. Data were anonymised and handled confidentially in accordance with data protection regulations. The present study used anonymised case reports; access to the original raw data remained with the respective student researchers.

## 3 Finding

The findings are presented using the four domains of the Person-Centred (Nursing) Practice (PC(N)PF) (5, 6, 15). Across these domains, the analysis illustrates how digital health technologies may both support and challenge person-centred practice in nursing homes. Claims reflect perceived contributions to person-centred care, whereas concerns capture tensions or limitations encountered in practice. The findings are derived from the narrative case reports and draw primarily on the perspectives of care professionals and family members, with more limited direct representation of residents' perspectives. Where available, observational material incorporated in the case reports also informed the findings. Specific case data and perspectives are identified using role specification (e.g., nurse, resident) and case numbering (C1-C3).

### 3.1 Person-centred outcomes

Within the PC(N)PF, expected outcomes relate to how care is experienced by residents, family and professionals, as well as the generation of a healthful culture (5, 6, 15). Across the three cases, technology-supported care was experienced in different ways across the perspectives represented. Professionals and family members often described reassurance and timely responses to care needs as valued aspects of technology-supported care. At the same time, there was recognition that technology-generated information requires contextualisation and may raise tensions around autonomy and relational proximity.

#### 3.1.1 Reassurance and timeliness

Across cases, professionals frequently linked technology use to reassurance and quicker responses. Technologies that enabled rapid access to information or visual verification were experienced as supporting faster assessment and prioritization. For example, being able to see what had happened helped determine appropriate actions: “*It is useful to know how someone fell and what happened beforehand*” (Elderly Care Physician, C1). In other accounts, time-based monitoring data supported a more nuanced understanding of the resident's condition, the duration of problems and the urgency for action (C1; C3).

Reassurance was also described by family members, particularly in situations of uncertainty. Family members described feeling less anxious knowing that staff could readily monitor situations, particularly at night. One relative described being more at ease: “*the sense of [*my relative*] being safeguarded gives me a very good feeling”* (C1). Another noted that it reduced the need to check on their loved one: “*Then I don't have to make a [*phone-*]call at night to ask whether everything is alright*” (C1). For some, monitoring was perceived as protective rather than restrictive: “*It is only good that she is being monitored; that is not a limitation of freedom*” (C1).

Timely access to and clarification of a resident's condition through remote diagnostics was similarly reassuring for professionals. Speed was explicitly appreciated: “*It is quite convenient that it [*the assessment*] can be done so quickly*.” (Physician, C2) One resident considered remote diagnostic technology acceptable when used competently and contributing to meaningful care: “*I actually find it quite normal… as long as it is done properly and something useful is done with it [*the findings*]”* (C2).

#### 3.1.2 Contextualised understanding

Across cases, technology-generated information was described as contributing to a more contextualised understanding of the residents' situation. For instance, some professionals described that monitoring night-time behaviour and sleep patterns enhanced understanding of behavioural patterns such as whether, when and how often residents left their bed (C3), supporting more tailored care decisions. Others felt that sleep is complex, personal and cannot be fully captured by sensor data outputs: “*[It's] very dry data about something that is not a dry topic at all, because so many factors play a role”* (Occupational therapist, C3). There was also recognition that technology-generated information requires contextual interpretation and cannot replace professionals' knowledge of the resident.

In practice, such data could support judgements about the urgency of a response. For example, indications of night-time agitation or frequent bed exits could prompt consideration of possible interventions, although staff emphasised that these should only be used as a last resort. When contextual information from staff or family was missing or not systematically documented, follow-up decisions could be limited (C3).

#### 3.1.3 Tensions around proximity and autonomy

Reassurance did not automatically translate into positive experiences for everyone. Across cases, tensions relating to autonomy and relational proximity were evident. Continuous or potential observation could conflict with a resident's sense of independence. One nurse recalled a resident saying: “*I am still independent; I simply don't want you to switch that camera on*” (C1).

Residents also described missing the physical presence of a doctor. Although remote assessment was efficient, some technology-mediated encounters were experienced as feeling more relationally distant than face-to-face contact (Observation data, C2). In Case 2, confidence in care relationships appeared to depend more on relational familiarity and continuity than on professional role: “*Sometimes I trust the nurse more, because I know her and see her [more] often than the doctor. The doctor doesn't know me the way my general practitioner used to”* (Resident, C2).

### 3.2 Care processes

Within the PC(N)PF, care processes relate to how professionals engage with residents and others in the delivery of care, including working with the person's values and preferences, communicating openly, and involving residents and family in decision-making (5, 6). Across the three cases, these processes became visible in how professionals explained and positioned the technologies, how they interpreted and responded to technology-generated information, and how decisions about technology use were discussed with residents and family members. These interactional practices varied in form and intensity across the cases.

#### 3.2.1 Sympathetic communication and expectation management

In the cases where the smart sensor and remote diagnostics were employed, professionals described efforts to position the technology as supporting rather than replacing human-based care. One care advisor explicitly framed the system as an aid: “*I always explain [*to the family*] that it is really a form of monitoring… that the technology supports the care*” (Care Advisor, C1).

In the case involving remote diagnostics, explanation was central to preparing residents and their families for what to expect, particularly when they were unfamiliar with the technology (C2). Nurses noted that although remote diagnostics could provide quicker assessment without a physician being physically present, this sometimes required careful explanation as part of a shared decision-making process: “'*So no doctor is coming then?’ that is the first thing they say… the mindset still has to change*” (Nurse, C2). In C2, informing and involving residents and family members in actions based on the results was described as supporting their sense of self-determination: “*As long as you get a message back. As long as they explain what is going on. That you know something*” (Resident, C2).

#### 3.2.2 Knowing the person mediates response to technology-generated information

In the smart sensor case, nurses described how knowing the resident and viewing the situation holistically helped them triage the urgency of their response to technology-generated information. Selectively checking and triaging information had become part of their everyday workflow. For example, one nurse explained: “*During care I sometimes choose not to look [*via the camera*]; I first see whose alert comes in, to make a quick assessment*” (Nurse, C1).

#### 3.2.3 Varied inclusion in decision-making processes

Across cases, involvement in decision-making varied. In the smart sensor case, the decision to install the technology had already been made independent of residents' or family members' perspectives. Professionals sometimes described its presence as part of the standard care environment in all resident rooms: “*It is as simple as a wall being pink. It is pink, and that device is on the ceiling. It is just there and there is nothing to change about it*” (Operational Advisor, C1).

In some situations, dialogue about the use of the technology did take place, particularly when concerns were raised about privacy (C1). However, in C1, accounts generally reflected limited resident and family participation in decision-making: “*They only asked me whether I agreed*” (Family Member, C1).

### 3.3 Care environment

Within the PC(N)PF, the care environment refers to the organisational and contextual conditions that shape how care is delivered, including teamwork, organisational support, and the physical and digital infrastructure (5, 6, 15). These conditions influence the way technology- and human-based care are enacted in everyday practice and consequently shape the lived experiences of those giving and receiving care. Across the three cases, digital health technologies use was closely intertwined with the care environment. This became visible in descriptions of shifting tasks and routines, the role of the physical environment, and the need for collaboration within and across professional teams.

#### 3.3.1 Shifting tasks, routines and conditions

Across the cases, technology-supported care was associated with changes in existing routines and working conditions. In the smart sensor case (C1), nurses reported that the system reduced the need for repeated physical checks on residents, which had previously required staff to walk down the hallway frequently and “*took time*” (Care assistant, C1). The technology was experienced by some as contributing to a calmer work atmosphere: “*The system does give more peace of mind compared to earlier alarm systems*.” (Nurse, C1) and it “…*saves many trips down the hallway and reduces panic situations*” (Care assistant, C1).

At the same time, the technology introduced new tasks around responding to alerts, adjusting settings and managing false alarms: “*No…(!) Report those false alerts*” (Nurse, C1). These additional activities could cause frustration, particularly when technical configurations were not retained. For example, alarm settings had to be manually reset at every change of shift: “*We now have to reset the settings for each resident after every night; that's not workable*” (Group interview, C1).

Working conditions were also influenced by technical reliability and infrastructural constraints. In the remote diagnostic case (C2), the physical environment was not always conducive to accurate measurement. Staff reported the challenge of finding quiet spaces so that the standard background noise could be eliminated and not affect the human sounds being measured: “*A lot of crackling… many sounds, but not what you hope to hear*” (Physician, C2). Alert accuracy and delayed support were other sources of frustration in cases C1 and C2, particularly digital connection, login problems, delayed starting-up and technical instability which “.. *drove me crazy*” (Nurse, C1), or waiting for technical support that “.. *take too long to be followed up*” (Nurse, C1).

In the remote diagnostics case (C2), technology use also affected professional roles and responsibilities. Physicians relied on nurses' observations and device-generated measurements when assessing residents remotely, which sometimes created hesitation due to uncertainty about the reliability of the recordings and the context in which they were obtained. At the same time, the use of remote diagnostics required nurses to collect and relay device-generated measurements, meaning that elements of clinical judgement became more distributed across professionals while formal medical responsibility remained with the physician. Staff shortages were reported to further complicated consistent use of the technology, particularly during evening shifts when uncertainty about who should initiate the technology and conduct follow-up actions delayed decision-making. Limited feedback from the system further reduced confidence in its use. As one nurse explained: “*I don't know what I did wrong… I don't get feedback from the device if the quality was good enough for the clinician*.” (Nurse, C2).

#### 3.3.2 Staff collaboration and effective utilisation

Across cases, an important aspect of the care environment concerned how technology-generated data were interpreted and used in practice. Although technologies provided technology-generated information, making sense of these data still required professional judgement and contextual knowledge.

This was particularly evident in the bed-based sensor case (C3), where large volumes of sleep-related data required active interpretation. Sensor output could not be viewed “*separately from the story alongside it*” and helped “*to map the situation more clearly*” (Nurse Practitioner, C3). Multidisciplinary discussions were therefore important for identifying underlying causes of flagged issues, as this “*makes a difference for the interventions that are ultimately chosen*” (Nurse Practitioner, C3).

In C3, interpretation of these data largely relied on professional judgement, as residents' own input was often limited due to cognitive impairments. In this case, multidisciplinary meetings remained the main setting in which the collected data were discussed and translated into care decisions, primarily involving professionals such as the nurse practitioner, psychologist and physician.

Findings from C3 further indicated that the technology's functionalities were not consistently embedded in nursing assessments, and contextual reporting (particularly during night shifts) was often limited. As a result, available data were not always complemented with contextual information about residents' behaviour or circumstances, restricting the depth of interpretation and subsequent care decisions.

### 3.4 Prerequisites

Within the PC(N)PF, prerequisites refer to the professional attributes staff bring to care relationships, including their values, beliefs, self-awareness and interpersonal competence (5, 6, 15). Across the cases, professionals generally valued technology-supported care when it supported professional competence and clinical judgement rather than replacing human judgement. Professionals' accounts also reflected moral awareness in relation to safety and privacy.

#### 3.4.1 Developing professional competence whilst using professional judgement

Professionals described gradually developing competence and confidence in technology-supported care as they became more familiar with the technologies and learned to use them in practice: “*It is nice to learn something new… and that it becomes easier over time. And that you can do it yourself*” (Nurse, C2). At the same time, concerns arose when technology-mediated care reduced direct face-to-face contact. In C2, where diagnostic assessment was conducted remotely through technology, caution was evident: “*I am certainly open to it, but I do need to be able to trust that what I hear… is correct* *..* *I notice that I am still quite sceptical because I don't yet dare to rely on it*” (Physician, C2). In C2, unexpected device responses were described as reducing professionals' confidence in the technology and increasing caution, particularly in the presence of family members.

Professionals generally viewed technology-based care as complementary to human-based care. Across the cases, the use of technology was not approached as a stand-alone decision-maker, but rather as an additional source of information that should be selectively interpreted alongside the professionals' knowledge of the individual resident. One occupational therapist stressed the need “*to get to know the person better… who someone really is*” (Occupational therapist, C3), and a nurse explained further how she selectively used the information gathered through the technology: “*I scan based on what I find important: I know the people*…” (Nurse, C1).

The data and visual outputs generated by technology had practical relevance when used to support clinical reasoning and decision-making. In C1, the speed with which clinicians could see what was happening was described as influencing decisions about referral or medication and prompting further assessment (e.g., delirium or urinary tract infections) (Physician, C1). Professionals also emphasised that technological information should remain complementary to human-based judgement. Human interpretation remained necessary, as technological data were described as “*Rather ‘dry’ data… something you have to be cautious with*.” (Group discussion, C3).

#### 3.4.2 Moral awareness

Professionals' accounts reflected ongoing consideration of values related to safety and privacy when technology-supported care was introduced. In the smart sensor case (C1), the system was installed in every resident's room. In one account, a nurse explicitly emphasized the importance of respecting residents' privacy: “*People simply have their own privacy*” (Nurse, C1). At the same time, other accounts suggested that the presence of the system was largely taken for granted in everyday practice. Within this context, dialogue with residents about their needs and wishes was described as an important starting point for discussing how the technology should be used: “*So especially seeking that dialogue [*about needs and desires*] together*” (Care advisor, C1).

Safety considerations were also emphasized, although professionals acknowledged that risks cannot be eliminated. As one physician noted: “*You can't eliminate all risks*…” (C1). Ethical reflection was not limited to residents' privacy alone. One psychologist described balancing residents' sense of privacy against staff's sense of safety during night shifts: “*I am always weighing these things… it also has to feel safe for the night shift who are there alone. So, it remains a bit of a seesaw*” (Psychologist, C1).

## 4 Discussion

### 4.1 Principle findings

This study explored how a person-centred lens offers new insights into technology-supported care in nursing homes. The findings suggest that the value of digital health technologies does not lie in the technologies themselves, but also how they are embedded in everyday care practices and relationships. Across the three cases, technologies were described as providing timely information, monitoring and additional insight into residents' situations, thereby informing care decisions and shaping interactions between professionals, residents and family members.

In practice, embedding technology in everyday care did not necessarily simplify care processes or the relational work involved in providing care. It often redistributed work by introducing new tasks, sometimes creating technical dependencies and at other times additional interpretive work for professionals (e.g., explaining the technology to residents and family members, managing alerts and system settings, dealing with login or connectivity problems, and interpreting sensor-generated data alongside contextual knowledge of the resident). As one researcher noted after reading the original case narratives: “*It's like the acceptance and rejection process in transplantation and implantation therapy: there's always a response, so careful screening and continued care are essential if a disastrous rejection is to be avoided*.”

### 4.2 Technology and person-centred care relationships

Our findings suggest that the way digital health technologies are experienced within person-centred care depends on how they are introduced and embedded in everyday practice. This is in line with the Person-Centred (Nursing) Practice Framework, which emphasises that person-centred outcomes emerge through the interaction between staff attributes (prerequisites), care processes and the care environment (5, 6, 15). For instance, across the cases, more positive experiences were described when professionals felt competent and confident in using the technology, when the care environment supported its use, and when residents and family members were informed and involved. Similar observations have been reported in studies showing that the value and meaning of digital health technologies emerge through their use in everyday care practices (16).

Our findings suggest that technology reshaped how professionals organised their work and engaged in care relationships. Monitoring data and alerts were often experienced as reassuring. However, interpreting and using these data was not always straightforward, as professionals needed to combine technological signals with their knowledge of residents and adapt their routines accordingly. In some cases, this also involved shifts in roles and responsibilities. In addition, our findings illustrate that technologies become meaningful when they are clearly explained, expectations are managed, and decisions about their use are discussed with residents and relatives. Similar observations have been reported in studies of digital and remote care technologies, which show that technological information only becomes meaningful when interpreted within care relationships and professional judgement (17, 18).

At the same time, tensions around privacy and autonomy also emerged, particularly in relation to monitoring technologies such as camera-based systems. Our findings indicate that, in some cases, relatives valued the sense of safety these technologies provided, whereas residents expressed discomfort with being monitored. This highlights a tension between residents’ preferences for autonomy and relatives’ best-interest considerations regarding safety, a dynamic commonly described in proxy decision-making in dementia care, where residents are not always able to fully articulate their preferences. Similar tensions between surveillance, safety and privacy have been reported in studies of monitoring technologies in long-term care (19, 20).

Particularly in nursing home settings, where many residents live with dementia, the introduction and use of technology require attentiveness to both the care environment into which the technology is introduced and integrated and the care processes that support sense-making and its meaningful acceptance or rejection. Together, these may contribute to positive care experiences and healthful cultures.

### 4.3 Reflecting on the silences and omissions in the narratives

During our analysis, the primary focus was on interpreting what was present in the case narratives rather than identifying what might be absent. Only after completing the analytical process did members of the research team begin to reflect on aspects that were not revealed in the case narratives.

From a hermeneutic perspective, attention to what remains unspoken can also contribute to understanding a phenomenon (21). When revisiting the case narratives through the lens of the PC(N)PF, several elements of person-centred practice appeared less visible, including residents’ values and beliefs in relation to technology-supported care, the authenticity of engagement between residents, families and professionals, and organisational aspects such as skill mix or power sharing. Their limited visibility does not necessarily mean that these elements were unimportant in practice, but may reflect the focus of the original case studies, which were not designed using the PC(N)PF as a guiding framework and therefore did not systematically explore these aspects.

The limited visibility of some person-centred elements may also relate to the context of nursing home care, where many residents live with dementia. In later stages of the condition, residents’ experiences and preferences are not always easily articulated or represented in research narratives. Direct resident participation was limited across the original studies, with residents included as interview participants only in C2. Consequently, the case narratives relied more strongly on the perspectives of professionals and family members and, in some cases, observational material. This limited direct representation of residents is particularly relevant when interpreting findings related to person-centred outcomes, autonomy and relational experience.

Several additional limitations should be considered when interpreting the findings. First, the secondary analysis was based on previously prepared narrative case reports rather than on a systematic re-analysis of the original interview and observational datasets. Consequently, the analysis depended on the selections, interpretations and narrative construction of the original studies, which may have limited the depth and traceability of some findings and increased the likelihood of silences or omissions. This limitation was compounded by the differing research questions of the original studies.

As some authors had been involved in the original case studies, providing contextual familiarity that supported interpretation but potentially also introducing confirmation bias. The involvement of researchers who had not participated in the original studies, together with critical peer dialogue, provided analytical distance, although these measures could not fully eliminate this potential source of bias.

Finally, the three cases were small-scale studies conducted in specific Dutch nursing home settings and involved technologically and ethically distinct forms of technology-supported care. The cross-case findings should therefore be understood as recurring relational and contextual patterns identified across these cases rather than as generalisable effects of digital health technologies in nursing homes. Nevertheless, the findings may resonate across comparable contexts and offer insights into fostering more person-centred approaches to technology-supported care in long-term residential care.

Recommendations at this stage include: (a) designing and testing reflective tools that examine how technologies are embedded in person-centred care practices, such as the Workplace Culture Critical Assessment Tool (WCCAT) (22); and (b) developing educational materials to encourage health and social care practitioners, as well as organisational leaders and change agents, to reconsider how technology-supported care is introduced and cultivated. From this perspective, success should not be measured by the quantity of technologies implemented, but by the appropriateness of their use as reflected in care experiences and the development of healthful cultures.

## 5 Conclusion

Viewing digital health technologies through a person-centred practice lens highlights that their value lies not only in the technologies themselves, but also in how they are introduced and integrated within everyday care practice and relationships. Across the cases examined, technology-supported care became meaningful through everyday care practices in which professionals interpret technological information, communicate with residents and relatives, and negotiate considerations such as safety, privacy and autonomy. The findings underline the importance of attending both to care processes and the care environment when introducing digital health technologies in nursing homes.

## Data Availability

The original contributions presented in the study are included in the article/Supplementary Material, further inquiries can be directed to the corresponding author.
